# Maize-soybean intercropping at optimal N fertilization increases the N uptake, N yield and N use efficiency of maize crop by regulating the N assimilatory enzymes

**DOI:** 10.3389/fpls.2022.1077948

**Published:** 2023-01-04

**Authors:** Jamal Nasar, Chang Jiang Zhao, Rayyan Khan, Hina Gul, Harun Gitari, Zeqiang Shao, Ghulam Abbas, Imran Haider, Zafar Iqbal, Waqas Ahmed, Raheela Rehman, Qing Ping Liang, Xun Bo Zhou, Juan Yang

**Affiliations:** ^1^ Guangxi Key Laboratory of Agro-environment and Agro-products Safety, National Demonstration Center for Experimental Plant Science Education, Agricultural College of Guangxi University, Nanning, China; ^2^ National Center of Industrial Biotechnology, Pir Mehr Ali Shah (PMAS) Arid Agriculture University, Rawalpindi, Pakistan; ^3^ Department of Agricultural Science and Technology, School of Agriculture and Environmental Sciences, Kenyatta University, Nairobi, Kenya; ^4^ College of Resources and Environmental Engineering, Jilin Institute of Chemical Technology, Jilin, China; ^5^ National Research Center of Intercropping, The Islamia University of Bahawalpur, Punjab, Bahawalpur, Pakistan; ^6^ Department of Plant Breeding and Genetics, University of Agriculture Faisalabad, Faisalabad, Pakistan; ^7^ Guangxi Agricultural Vocational University, Nanning, China

**Keywords:** maize-soybean intercropping, nitrogen yield, nitrogen use efficiency, nitrogen assimilatory enzymes, agricultural sustainability

## Abstract

**Introduction:**

Surplus use of chemical nitrogen (N) fertilizers to increase agricultural Q9 production causes severe problems to the agricultural ecosystem and environment. This is contrary to N use efficiency and sustainable agricultural production.

**Methods:**

Hence, this study was designed to investigate the effect of maizesoybean intercropping on N uptake, N yield, N utilization use efficiency, and the associated nitrogen assimilatory enzymes of maize crops under different N fertilization for two consecutive years 2021-2022.

**Results:**

The findings of the study showed that intercropping at the optimal N rate (N1) (250 kg N ha-1) increased significantly maize grain yield by 30 and 34%, residue yield by 30 and 37%, and 100-grain weight by 33 and 39% in the year 2021 and 2022, respectively. As compared with mono-cropping, at this optimal N rate, the respective increase (of maize’s crop N yield indices) for 2021 and 2022 were 53 and 64% for grain N yield, and 53 and 68% for residue N yield. Moreover, intercropping at N1 resulted in higher grain N content by 28 and 31%, residue N content by 18 and 22%, and total N uptake by 65 and 75% in 2021 and 2022, respectively. The values for the land equivalent ratio for nitrogen yield (LERN) were greater than 1 in intercropping, indicating better utilization of N under the intercropping over mono-cropping. Similarly, intercropping increased the N assimilatory enzymes of maize crops such as nitrate reductase (NR) activity by 19 and 25%, nitrite reductase (NiR) activity by 20 and 23%, and glutamate synthase activity (GOGAT) by 23 and 27% in 2021 and 2022, respectively. Consequently, such increases resulted in improved nitrogen use efficiency indices such as N use efficiency (NUE), partial factor nitrogen use efficiency (PFNUE), nitrogen uptake efficiency (NUpE), and nitrogen agronomic efficiency (NAE) under intercropping than mono-cropping.

**Conclusion:**

Thus, this suggests that maize-soybean intercropping under optimal N fertilization can improve the nitrogen status and nitrogen use efficiency of maize crops by regulating the nitrogen assimilatory enzymes, thereby enhancing its growth and yield. Therefore, prioritizing intercropping over an intensive mono-cropping system could be a better option for sustainable agricultural production.

## 1 Introduction

In China, there is increased use of chemical nitrogen (N) fertilizers for agricultural production, which results in wasted resources and environmental pollution (Malunga et al., 2018; Wang et al., 2018; Ahmad et al., 2022). For example, N leaching to subsoil increases soil acidification and groundwater pollution whereas its emission into the atmosphere directly stimulates air pollution (Galanopoulou et al., 2019). Such processes pose serious threats to the agricultural ecosystem and environment, contrary to efficient N use efficiency (NUE) (Galanopoulou et al., 2019; Nasar et al., 2020a; Nasar et al., 2020b). Also, intensive farming and long-term sole cropping system have severely harmed the agricultural ecology and reduced biodiversity (Nasar et al., 2020a). Thus, it is imperative to establish a sustainable agricultural production system that requires zero to little inputs. Hence, opting for intercropping over an intensive mono-cropping system could be a better option in such a scenario.

Intercropping is the simultaneous cultivation of two or more different crop species on the same field (Gitari et al., 2020; Maitra et al., 2020). It is an ancient agronomic practice and is still widespread globally. As opposed to mono-cropping, intercropping shows better growth and yield advantages due to the efficient utilization of the available natural resources (i.e., water, light, land, and nutrients) (Fung et al., 2019; Gao and Meng, 2020; Nasar et al., 2020b; Raza et al., 2021). Intercropping also helps in minimizing negative environmental impacts that threaten the agroecosystems (i.e., climate change, soil acidification, terrestrial eco-toxicity, or cumulative energy demand) (Yang et al., 2017; Nyawade et al., 2020b; Faridvand et al., 2021). In a cereal–legume intercropping, the companion crops efficiently utilize the atmospheric and soil available N. The major source of N under the such intercropping system is its fixation by the legumes, which helps save the soil N pool, increases the amount of soil N, enhances the N uptake in cereals and eventually crop yield (Xiang et al., 2018; Sousa et al., 2022). These improvements can occur through facilitative root interactions, nutrient sharing, and rhizosphere modification (e.g., enzymatic activities, root exudation, and soil pH) in an intercropping system (Li et al., 2019; Liu et al., 2019; Nasar et al., 2021). Such underlying mechanism under the intercropping systems contributes efficiently to soil nutrient cycling and plant nutrition (Nyawade et al., 2019; Nasar et al., 2020a). Additionally, the improved nitrogen assimilatory enzymes (i.e., NR, NiR and GOGAT activity) in the intercropping system equally contributes to the plant N content and its uptake (Nasar et al., 2022a). Previously, many studies have shown that cereal-legume cropping systems can significantly increase the plant N status due to the underlying rhizosphere modification (Sun et al., 2018; Nasar et al., 2020b; Raza et al., 2021), facilitative nutrient sharing through interspecific root interaction between intercrops (Shao et al., 2020) and improved N assimilatory enzymes (i.e., NR, NiR and GOGAT) (Nasar et al., 2022b).

Maize (*Zea mays* L) is grown globally due to its high-yielding food and forage crop production and is also known as the “Queen of Cereals” (Sun et al., 2014). In China, maize production increased by 1633% between 1949 and 2013, with average maize yields from 1 to 6 t ha^-1^ (Muhammad et al., 2022). More than 36 million hectares of maize were planted in the country in 2013, producing more than any other crop, especially on the North China Plain (Zhong et al., 2017). On the other hand, soybean (*Glycine max* L) is an annual grain legume known for its high protein content, vitamins, and minerals (Raza et al., 2020; Mirriam et al., 2022). It is a restorative plant that improves the quality and health of the soil by enriching it with nutrients (Zaeem et al., 2019). Thus, intercropping maize with soybean not only secures the regional food demand and nutritional quality of the forage industry but also improves the nutrient status of the maize crop besides providing an environmentally friendly and promising agricultural system for the future development. It is worth noting that, maize-soybean intercropping has been widely practiced to improve crop and forage yield, utilization of the natural resources, nutrient improvement of the cereal crop and soil health (Du et al., 2020; Raza et al., 2020; Nasar et al., 2022b). Nonetheless, relatively less data is available on the N yield, N use and utilization efficiency *via* regulation of N assimilatory enzymes in the maize-soybean intercropping. Therefore, this study was initiated to investigate the effect of maize-soybean intercropping on the N uptake, N yield, and N use efficiency, and the associated N assimilatory enzymes of maize with different N fertilization. The main objective of the study was to investigate whether maize-soybean intercropping under different N treatments improve the N yield, uptake and its use efficiency by regulating the N assimilatory enzymes of maize crop.

## 2 Material and methods

### 2.1 Site description, experimental design and layout

A two-year pot experiment was conducted at the experimental farm of Guangxi University, Nanning, China, in the year 2021-2022. This area is characterized by a subtropical monsoon climate with an annual rainfall of 1080 mm. The experimental site had soil with a loamy texture having an organic matter of 23.7 g kg^-1^, total N of 0.118%, alkaline N of 109.9 mg kg^-1^, available P of 73.6 mg kg^-1^, available K of 79.0 mg kg^-1^, soil pH of 7.4 and available iron of 97.7 mg kg^-1^.

**Figure 1:**
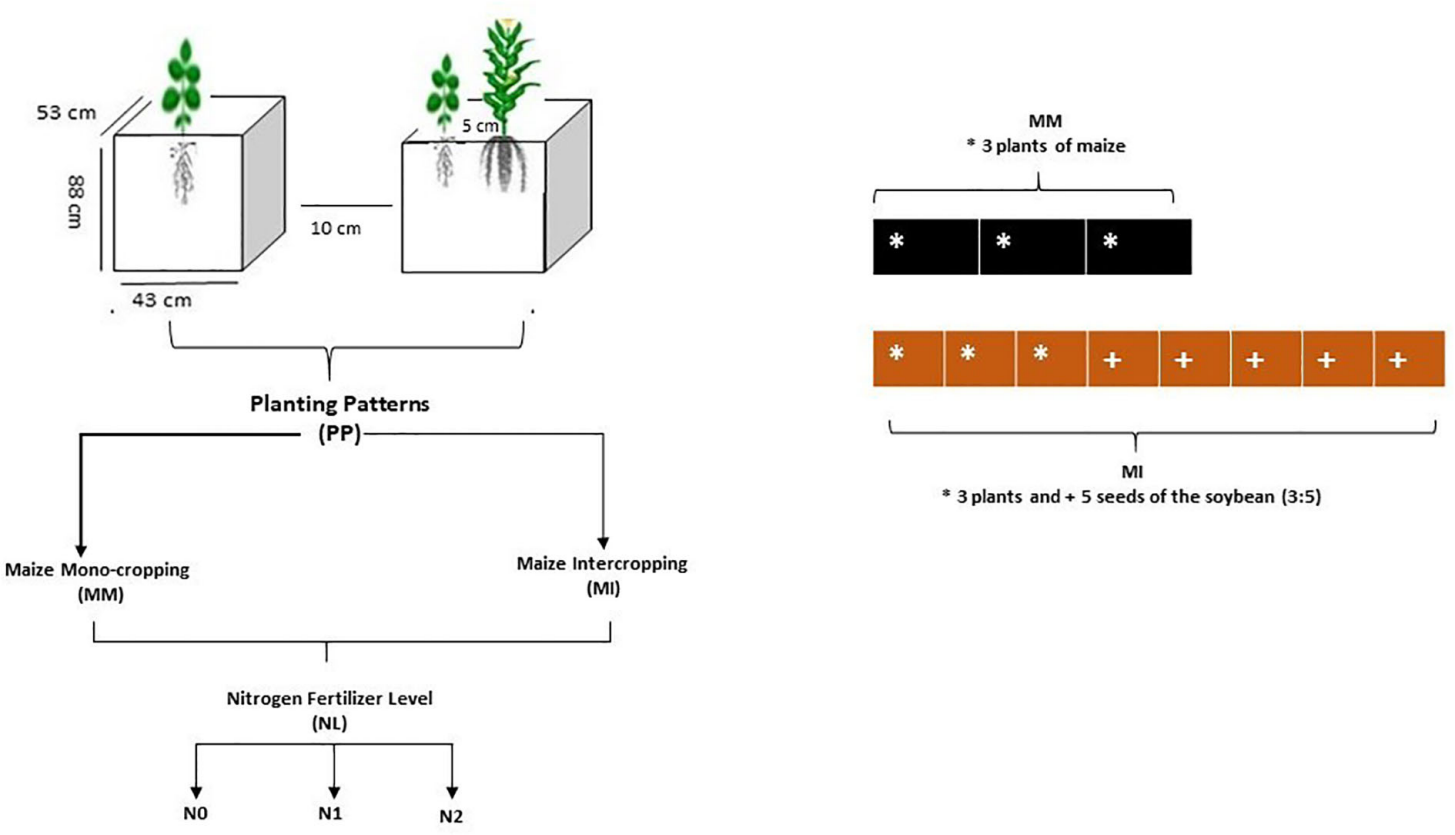
Schematic diagram of the experiment. N_0_; 0 kg N ha^-1^, N_1_; 250 N kg ha^-1^, N_2_; 300 kg N ha^-1^. * maize crop; + soybean crop.

Maize (Qing Qing 700 variety) was planted as a mono-crop (MM) and an intercrop (MI) with soybean (Gui Chun 15 variety) in large-sized pots (i.e., 88 cm height, 53 cm width, and 43 cm length) filled with 120 kg of soil. The pots, in four replicates, were randomly placed in a ventilated net house under natural light. Initially, five maize seeds and ten soybean seeds were planted in mono-cropping and intercropping at a plant density of 60,000 maize plants ha^-1^ and soybean seed rate of 20 kg seeds ha^-1^, respectively. However, later at the V3 growth stage, the maize and soybean plants were reduced to 3 and 5 (3:5) plants per pot, respectively, by uprooting the extra plants to better adapt to the pot environment (**Figure 1**). For the intercropping, maize and soybean plants were planted in the same pot such that the plant-to-plant and pot-to-pot distances were 5 and 10 cm, respectively. Additionally, the bottom of each pot was covered with small marble pebbles to minimize nutrient leaching. Planting and harvesting were done in mid-September 2021 and mid-February 2022, respectively for the first crop growing cycle, whereas the respective timings for the second cycle were mid-May 2022, and mid-October 2022.

**Figure 2:**
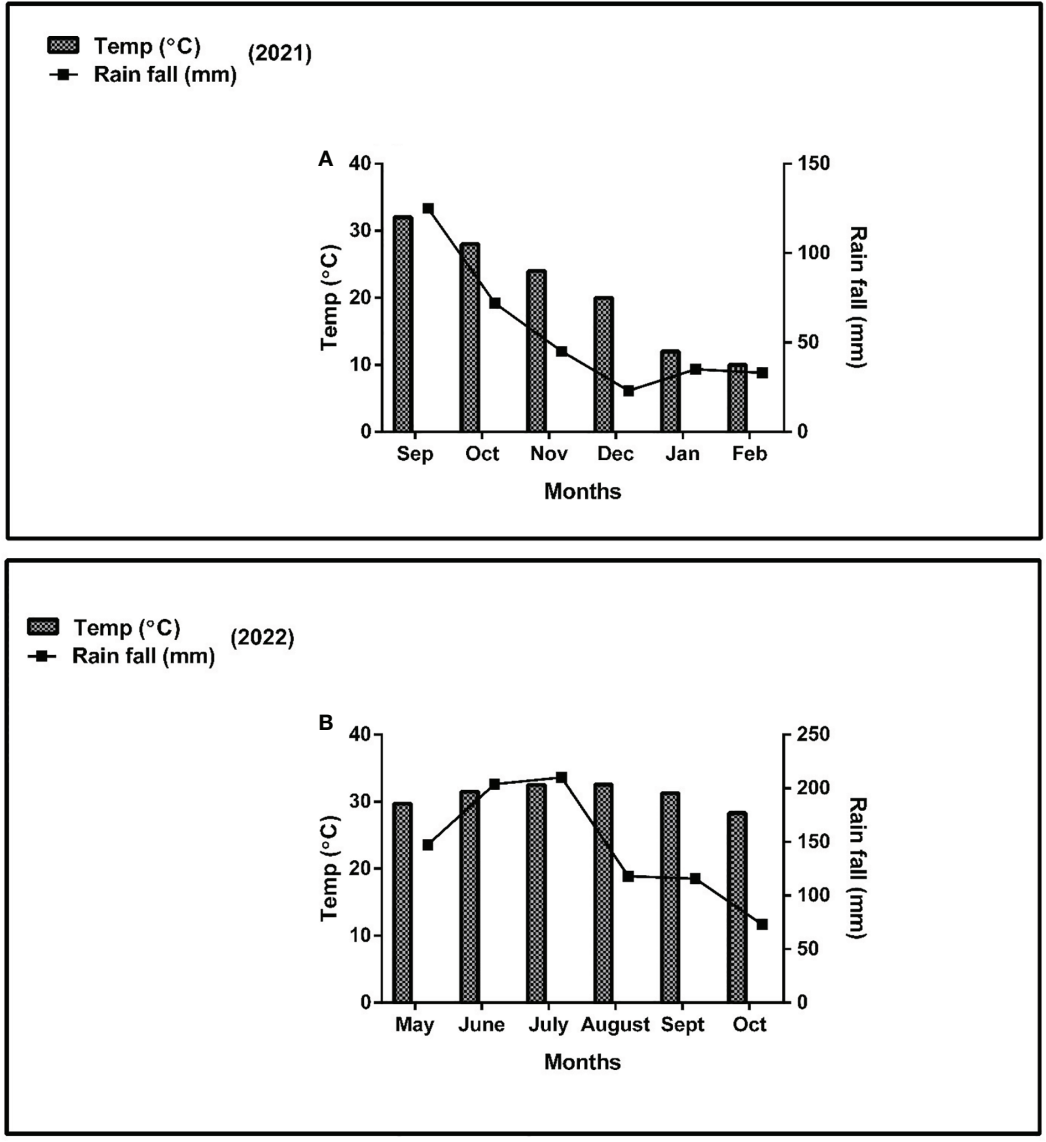
Weather forecast (temperature and rainfall) report of the experimental site during the experiment period **(A)**; year 2021 and **(B)**; year 2022.

Nitrogen fertilizer was applied as soil dressing before sowing at the rate of 0 kg N ha^-1^ (N_0_) for control, 250 kg N ha^-1^ (N_1_) for optimal and 300 kg N ha^-1^ (N_2_) for conventional practice. In addition, basal doses of phosphorus and potassium fertilizers were applied uniformly to all experimental pots (i.e., P at 100 kg ha^-1^ and K at 100 kg ha^-1^). The sources of fertilizers used were urea (46% N), diammonium phosphate (P_2_O_5_ 46% P), and potassium chloride (K_2_O 60% K). All the plants were watered normally, with weeds and insect pests being controlled with herbicides and pesticides, respectively, when needed. The environmental factors such as temperature (°C) and rainfall (mm) were carefully monitored and recorded (**Figures 2A, B**).

### 2.2 Data collection

#### 2.2.1 Grain and residue yield

The grain and residue yield of maize crops were obtained at full maturity when harvesting was done (Raza et al., 2020). The corn from maize crops was removed from the plant and threshed to determine 100-grain weight and grain yield by weighing them on an electric scale. After threshing, the remaining plant straw materials were sun-dried and oven-dried at 65°C for 72 h to obtain residue dry yield.

#### 2.2.2 Grain and residue N content and total N uptake

For the determination of grain and residue N content, the air and oven-dried plant samples were minced and passed through a 1 mm sieve. Nitrogen concentrations were determined as an average of duplicate samples of about 50 mg each by the Dumas combustion method (Neugschwandtner and Kaul, 2015) using an elemental analyzer (Vario MACRO cube CNS; Elementar Analysen-Systeme GmbH, Germany). The total N uptake was calculated as indicated in Equation 1 (Nasar and Shah, 2017).


(1) Total N uptake (g/pot)=(GNC ×Grain yield)+(RNC ×Residue yield)

Where GNC and RNC denote grain N content and residual N content, respectively.

#### 2.2.3 Nitrogen yield and nitrogen harvest index

The grain and residue N yield of maize crops were calculated as indicated in Equations 2 and 3 whereas, the N harvest index was computed according to Equation 4.


(2) Grain N yield (g/pot) =Grain yield ×Grain N content

(3) Residue N yield (g/pot) =Residue yield ×Residue N content 

(4) $$\text{N harvest Index }\left(\%\right)=\frac{\text{Grain N yield}}{\text{Residue N yield }}\times 100$$

#### 2.2.4 Nitrogen use efficiency indices

The nitrogen use efficiency (NUE), partial factor nitrogen use efficiency (PFNUE), nitrogen uptake efficiency (NUpE) and nitrogen agronomic efficiency (NAE) were calculated as indicated in Equations 5, 6, 7 and 8 (Sinebo et al., 2004; Anbessa and Juskiw, 2012).


(5) $$\text{NUE }\left({\text{g pot}}^{-1}\right)=\;\frac{\text{YLD}}{{\text{N}}_{\text{MIN}}+{\text{ N}}_{\text{f}}}\;$$

(6) $$\text{PFNUE }\left({\text{g pot}}^{-1}\right)=\;\frac{{\text{YLD}}_{\text{f}}}{{\text{N}}_{\text{f}}}$$

(7) $$\text{NUpE }\left({\text{g pot}}^{-1}\right)=\;\frac{\text{total N uptake}}{{\text{N}}_{\text{MIN}}+{\text{ N}}_{\text{f}}}$$

(8) $$\text{NAE }\left({\text{g pot}}^{-1}\right)=\;\frac{\text{grain N YLD}-\text{grain YLD}}{{\text{N}}_{\text{MIN}}+{\text{ N}}_{\text{f}}}$$

Where the YLD is the grain yield and NY_r_ is the residue nitrogen yield of maize crops; N_MIN_ represents soil mineral N at sowing and N_f_ the fertilizer level; the subscript f stands for fertilizer N.

#### 2.2.5 Nitrogen assimilatory enzymes

The nitrogen assimilatory enzyme activity, such as nitrate reductase (NR), nitrite reductase (NiR), and glutamate synthase (GOGAT) activity in maize leaf samples were determined according to the following protocol.

##### 2.2.5.1 NR activity

To determine NR activity in maize leaves, the frozen plant leaf samples were crushed in 4 mL of 25 mM sodium phosphate (buffered at pH 8.7) containing 1.3 mM EDTA and 10 mM cysteine before being centrifuged at 4000 rpm for 15 minutes at 4°C. In this case, the reaction mixture was made up of 0.1 M KNO_3_, and 2.82 mM NADH. Following addition of NADH was a 30-minute incubation period. After 15 minutes, the reaction was stopped followed by addition of 1% sulfanilamide and 0.02% N-phenyl-2-naphthylamine. The absorbance was the calculated at 540 nm following centrifugation at 4000 rpm for 5 min (Imran et al., 2019).

##### 2.2.5.2 NiR activity

NiR activity (NiR, EC 1.7.2.1) in the fresh maize leaves was determined according to the proposed method of Rao et al. (1981). Briefly, a cold 0.1 M potassium phosphate (buffered at a pH of 7.5) was used to homogenize the frozen leaf tissues. The reaction mixture included enzyme extract, 10 mM KNO_2_, 1.5% methylviologen, and 5% sodium dithionite (Na_2_S_2_O_4_) dissolved in 100 mM NaHCO_3_, which was added to start the reaction. The 30-minute incubation period of the reaction mixture at room temperature was followed by methylviologen’s decolorization. Nitrite concentrations were determined by measuring the absorbance at 540 nm in a solution made up of supernatant, distilled water, 1% (w/v) N (1-naphty1)-ethylenediamine dihydrochloride, and 10% (w/v) sulfanilamide produced in HCl.

##### 2.2.5.3 GOGAT activity

The NADH-glutamate synthase (NADH-GOGAT; EC 1.4.1.14) activity in maize leaves was measured according to Lin and Kao (1996). In this case, frozen leaves were homogenized in a mortar and pestle with an extraction buffer that was pre-cooled and containing 100 mM Tris-HCl (pH 7.6), 1.0 mM MgCl_2_-6H_2_O, 10 mM 2-mercaptoethanol, and 1.0 mM ethylenediaminetetraacetic acid (EDTA). The homogenates were centrifuged for 15 minutes at 4°C at 13,000 rpm. To evaluate the GOGAT enzymes in leaf tissues, the supernatants were used as crude extracts. 25 mM Tris-base, 100 mM -Ketoglutaric acid, 10 mM KCl, 20 mM L-glutamine, and 3 mM NADH were used to treat the crude enzyme extract. Thereafter, NADH oxidation caused the absorbance which was measured at 340 nm.

#### 2.2.6 Land equivalent ratio for nitrogen yield (LER_N_)

The land equivalent ratio for nitrogen yield (LER_N_) as an indicator used to determine the N yield advantage of intercrops (Mead and Willey, 1980) was calculated as shown in Equation 9:


(9) $${\text{LER}}_{\text{N}}=\;\frac{{\text{NY}}_{\text{MI}}}{{\text{NY}}_{\text{MM}}}$$

Where NY_MI_ and NY_MM_ are the crop N yields for maize under intercropping and mono-cropping, respectively. A LER_N_ > 1, indicates a higher N yield whereas when its<1 then it represents a lower N yield.

### 2.3 Statistical analysis

The collected data were entered and tabulated in Ms excel 2016. For statistical analysis, two factors factorial analysis was done using the SPSS and Ms statistix 6.1 statistical analysis software, respectively. Means among the treatments were compared by Least Significant Difference (LSD) Test at *p* ≤ 0.05 level of probability (Mirzapour et al., 2022) by keeping the nitrogen fertilization as the main effect and planting pattern sub-effect. Graphs were constructed using the graphical software Graph Pad prism 6.1.

## 3 Results

### 3.1 Grain and residue yield

Intercropping and N fertilization significantly (*p<* 0.05) affected the grain yield, residue yield and 100-grain weight of maize (**Table 1**). However, these indices were more evident in intercropping under N_1_ treatment than in N_0_ and N_2_ treatments. For instance, in 2021, intercropping increased the grain yield of maize crops by 16, 30 and 20% in N_0_, N1 and N_2_, respectively compared with mono-cropping, whereas in 2022, the respective increases of 18, 34 and 19% in 2022 were noted. Moreover, intercropping increased the residue yield of maize crops by 15, 30, and 24% in 2021 and by 19, 37 and 23% compared with mono-cropping. Similarly, intercropping increased the 100-grain weight of maize crops by 3% under N_1_ treatment than by 23 and 26% and by 26 and 39 and 29% under N_0_ and N_2_ treatments in 2021 and 2022, respectively when compared with mono-cropping.

**Table 1:** Grain yield, residue yield and 100-grain weight of maize crop as influenced by different planting patterns (MM, maize mono-cropping and MI, maize intercropping) and N fertilizer application rates (N_0_; 0 _kg_ N ha^-1^, N_1_; 250 kg N ha^-1^ and N_2_; 300 kg N ha^-1^) in 2021 and 2022 crop growing seasons.

Treatment		Grain yield (g pot^-1^)		Residue yield (g pot^-1^)		100-grain weight (g)	
N fertilizer rate	Plantingpattern	Year 2021	Year 2022	Year 2021	Year 2022	Year 2021	Year 2022
**N_0_**	**MM**	91.50 ± 8.1 d	93.00 ± 786.0 d	201.36 ± 9.5 d	205.61 ± 854.2 c	22.52 ± 1.6 c	22.13 ± 2.0 d
	**MI**	106.18 ± 11.6 c	109.94 ± 810.1 c	230.64 ± 7.4 c	244.89 ± 781.8 b	27.66 ± 2.1 b	27.90 ± 3.1 bc
**N_1_**	**MM**	105.15 ± 4.5 c	105.90 ± 621.4 c	210.40 ± 10.1 d	213.15 ± 750.8 c	27.81 ± 2.1 b	27.83 ± 2.3 c
	**MI**	136.54 ± 2.9 a	142.29 ± 707.0 a	272.80 ± 10.6 a	291.30 ± 855.2 a	37.09 ± 1.7 a	38.71 ± 3.2 a
**N_2_**	**MM**	102.05 ± 8.1 cd	103.80 ± 621.4 cd	204.15 ± 8.5 d	207.15 ± 750.8 c	23.81 ± 1.1 c	25.08 ± 3.1 cd
	**MI**	122.62 ± 3.2 b	123.37 ± 707.0 b	253.55 ± 17.7 b	254.80 ± 855.2 b	30.10 ± 0.6 b	32.46 ± 1.9 b
**Significance**							
**NL**		0.000***	0.000***	0.003**	0.001**	0.001**	0.000***
**PP**		0.000***	0.000***	0.000***	0.000***	0.000***	0.000***
NL*PP		0.150^ns^	0.061^ns^	0.065^ns^	0.020*	0.106 ^ns^	0.269 ^ns^

The mean values with different lowercase letters (± standard deviation) are significantly different from each other at LSD Test (P ≤ 0.05). *p< 0.05, **p< 0.01, ***p< 0.001, ^ns^ p > 0.05.

### 3.2 N yield indices and N harvest index

The collected data showed that intercropping under different N fertilization significantly (*p<* 0.05) increased the N yield indices of the maize crop as compared with mono-cropping (**Table 2**). However, these indices were more pronounced under N_1_ than in N_0_ and N_2_ treatments. In 2021, intercropping increased the grain N yield of maize by 53% under N_1_ treatment than by 27 and 39% under N_0_ and N_2_ treatments, respectively when compared with mono-cropping. There was a further increase in the grain N yield of maize crop under intercropping of 64% in N_1_ vis a vis 32 and 40% under N_0_ and N_2_, respectively in 2022 as compared with mono-cropping. Similarly, when compared with mono-cropping, intercropping significantly (*p*< 0.05) increased the residue N yield of maize crops by 53% under N_1_ treatment than by 25 and 43% under N_0_ and N_2_ treatments, respectively in 2021, and further increased by 68% under N_1_ treatment than by 33 and 44% under N_0_ and N_2_ treatments, respectively in 2022. However, the N harvest index of maize crops did not show any significant differences in the intercropping under N fertilization treatments. In addition, the LER_N_ was always greater than 1 in all N treatments, indicating a yield advantage of intercropping with N treatments.

**Table 2:** Grain N yield, residue N yield, N harvest index and LER_N_ of maize crop as influenced by different planting patterns (MM, maize mono-cropping and MI, maize intercropping) and N fertilizer application rates (N_0_; 0 _kg_ N ha^-1^, N_1_; 250 kg N ha^-1^ and N_2_; 300 kg N ha^-1^) in 2021 and 2022 crop growing seasons.

Treatment		Grain N yield (g pot^-1^)		Residue N yield(g pot^-1^)		N harvest index (%)		LER_N_	
N fertilizer rate	Plantingpattern	Year 2021	Year 2022	Year 2021	Year 2022	Year 2021	Year 2022	Year 2021	Year 2022
**N_0_**	**MM**	203.70 ± 19.4 d	208.17 ± 19.6 d	448.66 ± 28.0 e	460.75 ± 28.9 d	45.63 ± 6.1	45.36 ± 5.5		
	**MI**	259.05 ± 35.8 c	275.43 ± 29.8 c	562.08 ± 24.1 c	613.49 ± 31.4 c	45.98 ± 5.1	44.85 ± 3.7	1.3	1.3
**N_1_**	**MM**	263.76 ± 16.7 c	262.60 ± 21.5 c	527.42 ± 28.9 cd	527.76 ± 14.3 d	50.06 ± 3.2	49.73 ± 3.5		
	**MI**	403.88 ± 32.9 a	431.69 ± 34.3 a	807.54 ± 80.2 a	884.07 ± 65.8 a	50.08 ± 1.1	48.87 ± 2.7	1.5	1.6
**N_2_**	**MM**	241.54 ± 18.8 cd	241.50 ± 21.7 cd	484.35 ± 37.4 de	482.68 ± 38.1 d	50.15 ± 6.1	50.26 ± 5.9		
	**MI**	336.24 ± 28.6 b	338.45 ± 29.1 b	694.58 ± 72.1 b	697.15 ± 66.7 b	48.55 ± 3.5	48.81 ± 5.7	1.4	1.4
**Significance**									
**NL**		0.000***	0.000***	0.000***	0.000***	0.164^ns^	0.133^ns^		
**PP**		0.000***	0.000***	0.000***	0.000***	0.830^ns^	0.631^ns^		
NL*PP		0.017*	0.003**	0.013*	0.000***	0.901^ns^	0.979^ns^		

The mean values with different lower case letters (± standard deviation) are significantly different from each other at LSD Test (P ≤ 0.05). *p< 0.05, **p< 0.01, ***p< 0.001, ^ns^ p > 0.05.

### 3.3 N concentration and total N uptake

**Figure 3:**
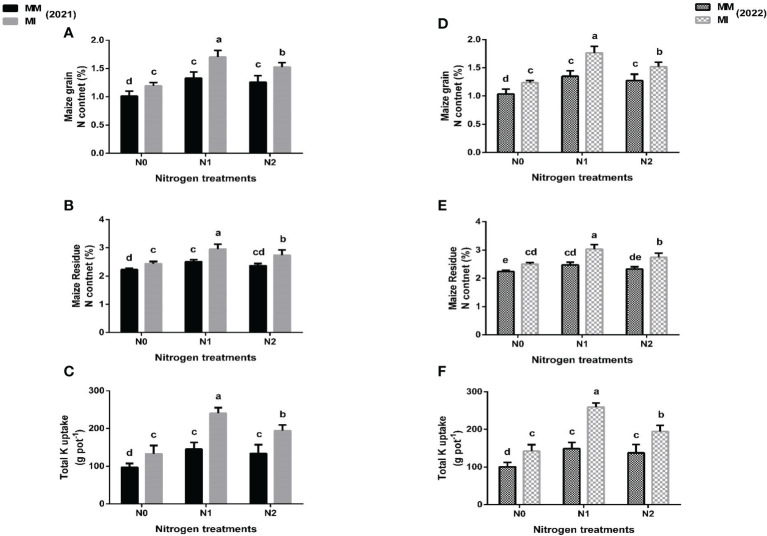
Maize grain N content **(A, D)**, residue N content **(B, E)**; total N uptake **(C, F)** under different planting patterns (MM, maize mono-cropping and MI, maize intercropping) and N fertilizer application rates (N_0_; 0 _kg_ N ha^-1^, N_1_; 250 kg N ha^-1^ and N_2_; 300 kg N ha^-1^) in 2021 and 2022 crop growing seasons. The column bars with dissimilar lowercase letters are significantly different from each other as per the LSD test (*p*< 0.05).

When compared with mono-cropping, intercropping significantly (*p*< 0.05) enhanced the N concentration and total N uptake of maize crops under different N fertilization treatments (**Figure 3**). In 2021, intercropping significantly (*p*< 0.05) increased the N concentration of maize grain and residues by 28 and 18% under N_1_ treatment than by 18 and 9% in N_0_, and by 21 and 16% in N_2_ respectively as compared with mono-cropping (**Figures 3A, B**). In 2022, intercropping further increased the N concentrations of maize grain and residue by 31 and 22% under N_1_ than by 20 and 12% in N_0_, and by 19 and 18% in N_2_, respectively as compared with mono-cropping (**Figures 3D, E**). Moreover, intercropping increased the total N uptake of maize crop by 65% under N_1_ vis a vis 37 and 45% in N_0_ and N_2_, respectively in 2021. Nonetheless, higher increases were noted in 2022. For instance, there was 75% increase in N uptake in the intercropping under N_1_ than by 41 and 32% in N_0_ and N_2_, respectively when compared with mono-cropping (**Figures 3C, F**).

### 3.4 Nitrogen use and utilization

**Figure 4:**
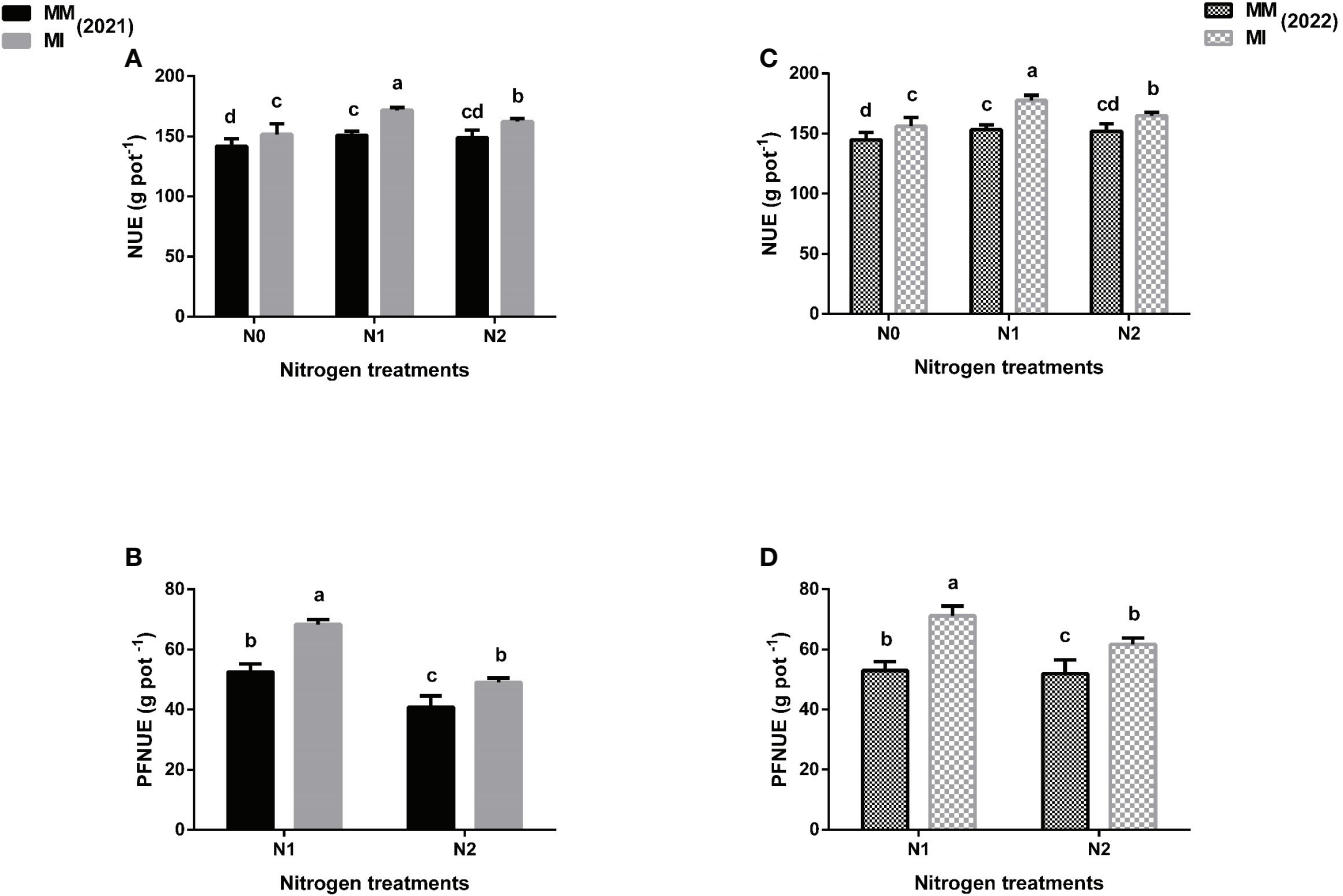
Maize nitrogen use efficiency (NUE) **(A, C)**, partial factor nitrogen use efficiency (PFNUE) **(B, D)** under different planting patterns (MM, maize mono-cropping and MI, maize intercropping) and N fertilizer application rates (N_0_; 0 _kg_ N ha^-1^, N_1_; 250 kg N ha^-1^ and N_2_; 300 kg N ha^-1^) in 2021 and 2022 crop growing seasons. The column bars with dissimilar lowercase letters are significantly different from each other as per the LSD test (*p*< 0.05).

**Figure 5:**
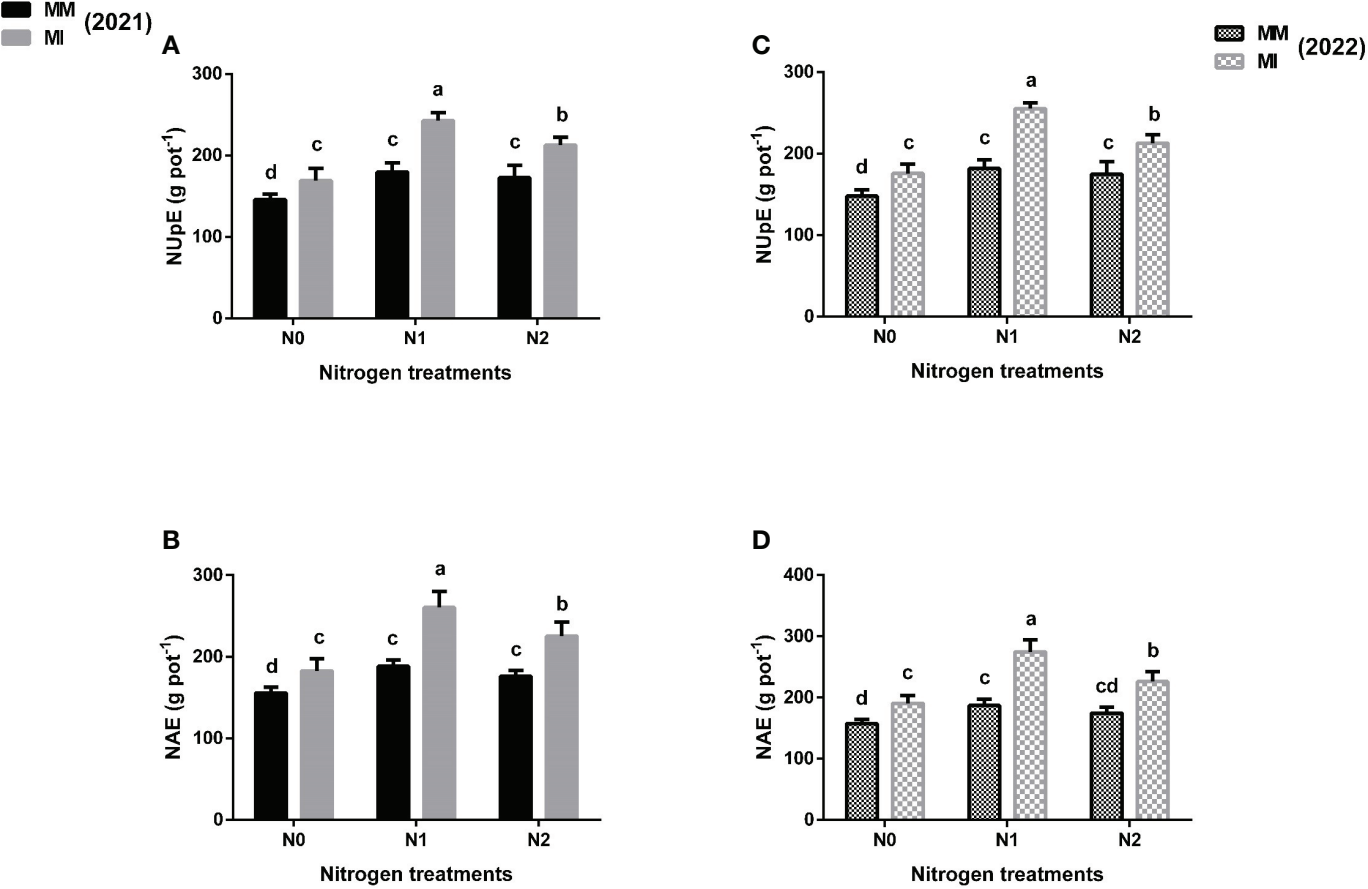
Maize nitrogen uptake efficiency (NUpE) **(A, C)**, nitrogen agronomic efficiency (NAE) **(B, D)** under different planting patterns (MM, maize mono-cropping and MI, maize intercropping) and N fertilizer application rates (N_0_; 0 _kg_ N ha^-1^, N_1_; 250 kg N ha^-1^ and N_2_; 300 kg N ha^-1^) in 2021 and 2022 crop growing seasons. The column bars with dissimilar lowercase letters are significantly different from each other as per the LSD test (*p*< 0.05).

Intercropping and N fertilization significantly affected the nitrogen use efficiency indices such as NUE, PFNUE, NUpE and NAE of maize crops when compared with mono-cropping (**Figures 4**, **5**). However, these indices were more promising in intercropping under N_1_ treatment than in N_0_ and N_2_ treatments. In 2021, intercropping significantly (*p*< 0.05) increased the NUE by 14% under N_1_ treatment than by 7 and 9% under N_0_ and N_2_, respectively when compared with mono-cropping (**Figures 4A, C**). In 2022, there was an increase of 16% for this index under intercropping for N_1_ vis a vis 8 and 9% for N_0_ and N_2_, respectively. Moreover, intercropping increased the PFNUE of maize crops by 30 and 34% under N_1_ treatments than by 20% and 19% under N_2_ treatments in 2021 and 2022, respectively as compared with mono-cropping (**Figures 4B, D**). Similarly, intercropping increased the NUpE of maize crop by 35% under N_1_ treatment than by 16 and 23% under N_0_ and N_2_ treatments respectively in 2021, and further increased by 40% under N_1_ treatment than by 19 and 22% under N_0_ and N_2_ treatments, respectively in 2022 as compared with mono-cropping (**Figures 5A, C**). Furthermore, intercropping increased the NAE by 38% under N_1_ treatment than by 17 and 28% under N_0_ and N_2_ treatments in 2021, and further increased by 47% under N_1_ treatment than by 21 and 29% under N_0_ and N_2_ treatments respectively in 2022 as compared with mono-cropping (**Figures 5B, D**).

### 3.5 Nitrogen assimilatory enzymes

**Figure 6:**
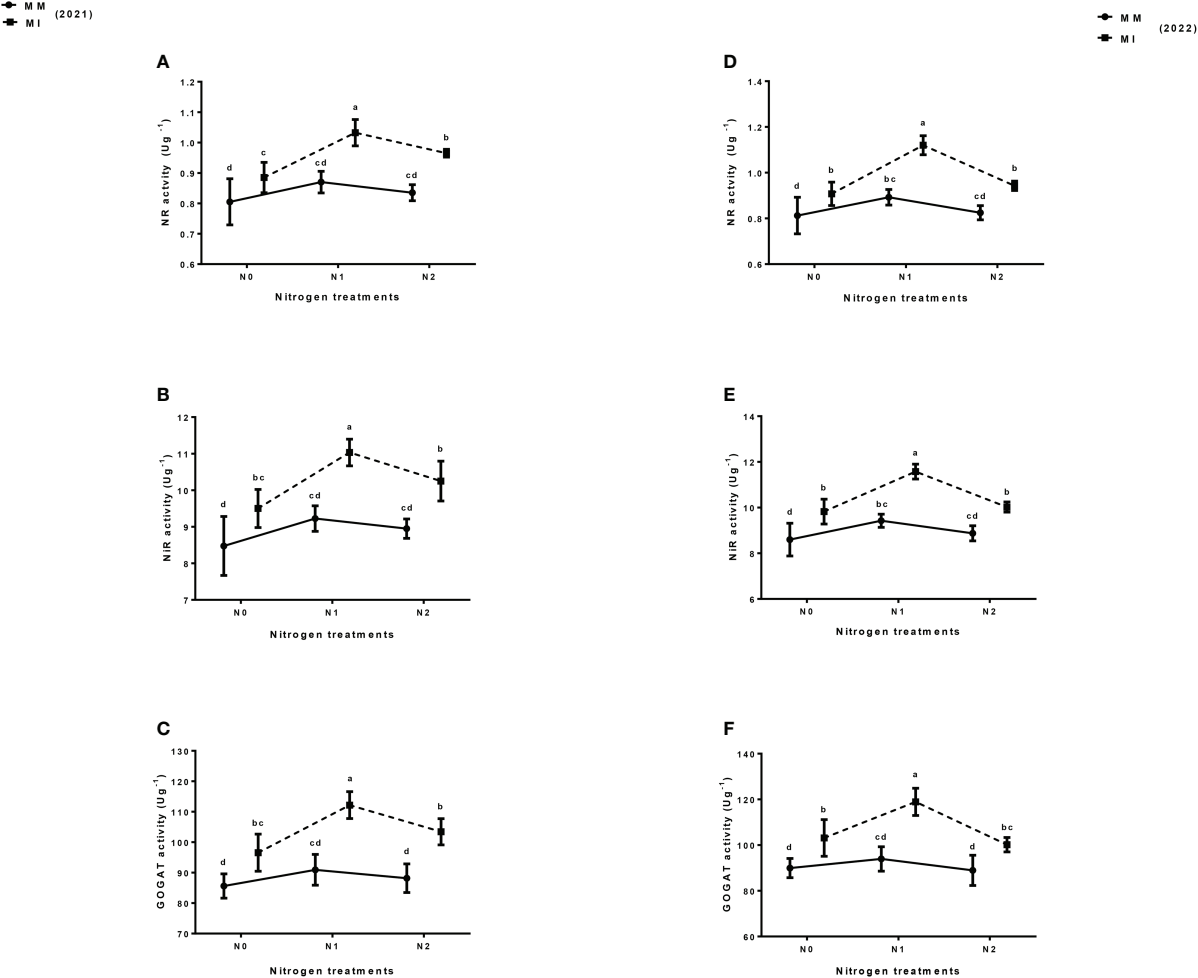
Maize nitrogen assimilatory enzymes: nitrate reductase (NR) **(A, D)**, nitrite reductase (NiR) **(B, E)** and glutamate synthase (GOGAT) **(C, F)** activity under different planting patterns (MM, maize mono-cropping and MI, maize intercropping) and N fertilizer application rates (N_0_; 0 _kg_ N ha^-1^, N_1_; 250 kg N ha^-1^ and N_2_; 300 kg N ha^-1^) in 2021 and 2022 crop growing seasons. The column bars with dissimilar lowercase letters are significantly different from each other as per the LSD test (*p*< 0.05).

Intercropping and nitrogen fertilization significantly (*p*< 0.05) affected the nitrogen assimilatory enzymes of maize as compared with mono-cropping. For instance, compared with mono-cropping, intercropping increased the NR, NiR and GOGAT activity of maize crop under different N treatments (**Figure 6**). However, these activities were more enhanced under N_1_ treatment than in N_0_ and N_2_ treatments. In 2021, the NR activity of maize crops increased by 19% under N_1_ than by 10 and 16% under N_0_ and N_2_, respectively, but this activity was further increased by 25% in intercropping system under N_1_ treatment than by 12 and 14% under N_0_ and N_2,_ respectively in 2022 as compared with mono-cropping (**Figures 6A, D**). Similarly, intercropping increased the NiR activity of maize crops by 20% under N_1_ treatment than by12 and 15% under N_0_ and N_2_, respectively in 2021, but it was further increased by 23% in the intercropping system under N_1_ treatment than by 14 and 13% under N_0_ and N_2_, respectively in 2022 as compared with mono-cropping (**Figures 6B, E**). Moreover, intercropping increased the GOGAT activity of maize crop by 23% under N1 treatment than by 13 and 17% under N_0_ and N_2_ treatments respectively in 2021, and further increased by 27% under N_1_ treatment than by 15 and 13% under N_0_ and N_2_ treatments respectively in 2022 when compared with mono-cropping (**Figures 6C, F**).

### 3.6 Liner regression

**Figure 7:**
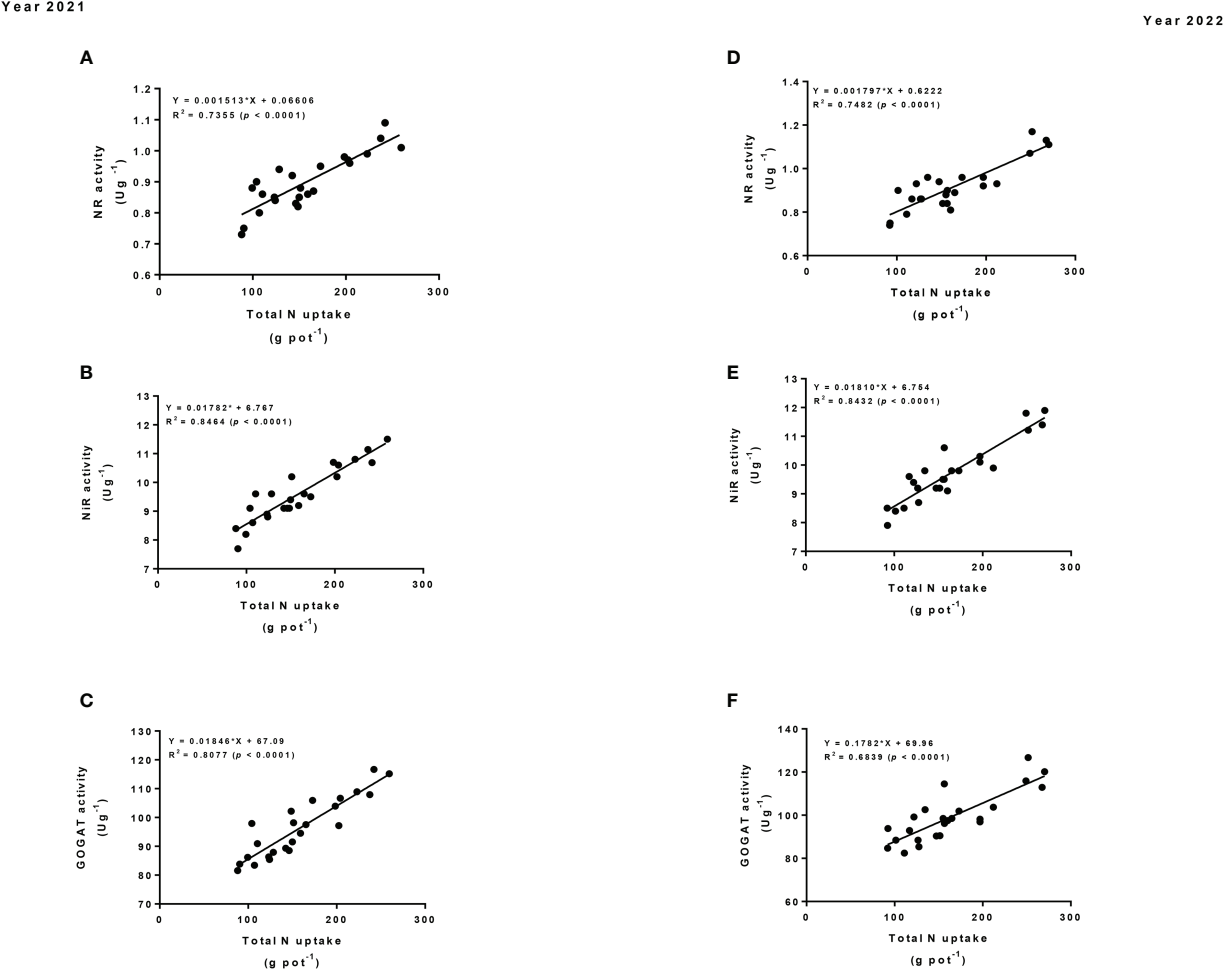
Regression analysis of the total N uptake with nitrogen assimilatory enzymes (i.e., NR **(A, D)**, NiR **(B, E)**, and GOGAT activity **(C, F)**) of maize crop in 2021 and 2022.

**Figure 8:**
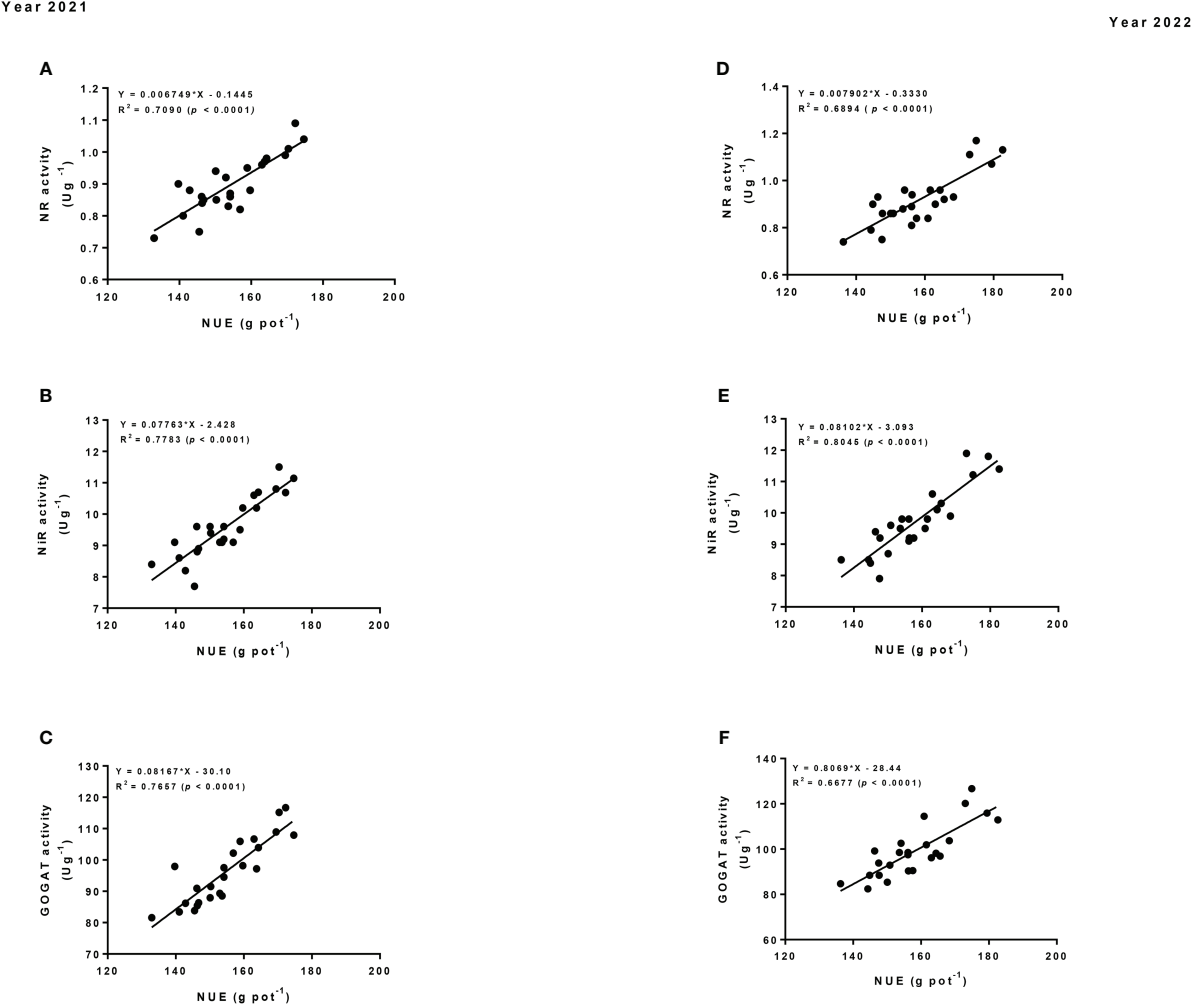
Regression analysis of nitrogen use efficiency (NUE) for maize crop with nitrogen assimilatory enzymes (i.e., NR **(A, D)**, NiR **(B, E)**, and GOGAT activity **(C, F)**) in 2021 and 2022.

The linear regression analysis was used to determine the relationship of the total N uptake and NUE with the N assimilatory enzymes (i.e., NR, NiR and GOGAT activity) of maize crop. The result showed that the total N uptake had significant strong correlations with NR, NiR and GOGAT activity (**Figures 7A–F**). Equally, NUE had significant positive and strong correlations with NR, NiR and GOGAT activity (**Figures 8A–F**). Thus, such relationships suggested that changes in the N assimilatory enzymes could significantly bring changes in the total N uptake and NUE of the maize crop under intercropping.

## 4 Discussion

Generally, the improved productivity of intercropping is due to the efficient utilization of the available resources (e.g., water, nutrients, land and light) (Neugschwandtner and Kaul, 2015; Rawashdeh, 2016; Gitari et al., 2018a; Gitari et al., 2018b; Gou et al., 2018; Raza et al., 2019). The present study demonstrated that maize-soybean intercropping significantly increased the yield indices, residue yield and 100-grain weight of maize crop. However, these indices were more evident under N_1_ treatment than N_0_ and N_2_. Possibly, this could due to the better utilization of the available natural resources such as land, light, water, and nutrients (Nasar et al., 2020b; Raza et al., 2020; Raza et al., 2022), or could be due to the N fertilization, which is an important element required for plant growth and development (Zhang et al., 2014; Nduwimana et al., 2020). Moreover, legume in intercropping with cereal are also known to improve the N status of cereal crop by facilitative transfer of N to their corresponding cereal crop through the underlying facilitative root interactions, which ultimately leads to an increase yield production of intercropping cereals than mono-cropping (Shao et al., 2020). As previously documented that the efficient use of the available resources (i.e., water, land, light and nutrients) by intercrops have produced more yield than in their mono-cropping system (Latati et al., 2017; Raza et al., 2019; Kisaka et al., 2023). Maize/mungbean intercropping have also shown to increase the grain yield and biomass dry matter of maize crop by 15-29% and 21-34%, respectively than in mono-cropping, which was attributed to better utilization of the available resources and the underlying nutrient sharing of mungbean to its corresponding maize crop during intercropping (Qian et al., 2018). Moreover, maize in intercropping with mungbean or mash bean significantly enhance the yield and biomass dry matter of maize crops particularly under optimal N fertilization, mainly because of the N fixation ability of legumes, which helps improve the N content of maize crop. This helps in reducing the high use of chemical N fertilizers (Saleem et al., 2011), which supports our findings. Intercrops are also known for their better use of the applied fertilizers, which helps in production of more crop yield under intercropping than in mono-cropping systems that are established under the same piece of land with same or different fertilization managements (Shao et al., 2020). For instance, the higher LER_N_ value (1.33) in oat-pea intercropping than in mono-cropping under optimal N fertilization was mainly because of the better utilization of the applied N fertilizer (Neugschwandtner and Kaul, 2015; Sun et al., 2018). Similarly, in our study we found a higher LER_N_ values in intercropping than in mono-cropping, indicating better utilization of the N in the intercropping system than in mono-cropping. Previously different intercropping studies have shown higher LER_N_ value under optimal N fertilization (Neugschwandtner and Kaul, 2015; Sun et al., 2018), which confirmed our results.

Legumes are well known for their ability to fulfill nitrogen requirement through atmospheric N fixation. Thus, legumes in intercropping with cereals can help improve the N content and its uptake by cereals due to the underlying facilitative N transfer through interspecific root interaction (Nasar et al., 2020b; Raza et al., 2020; Shao et al., 2020; Mirriam et al., 2022). In our study we found that soybean when intercropped with maize significantly increased the N content and total N uptake of maize crops than in mono-cropping. However, these indices were more prominent under N_1_ treatment than in N_0_ and N_2_ treatment. There could be several reasons to explain such observation, (i) this could be due the N fixation ability of legume which improved the soil nutrient pool and N availability, thereby enhancing the N content and its uptake of the cereal crop during intercropping (Neugschwandtner and Kaul, 2015; Sousa et al., 2022), (ii) it could also be attributed to the underling nutrient sharing between intercrops or facilitative N transfer from legumes to their corresponding cereal crop (Zhang et al., 2017a; Shao et al., 2020), (iii) it might also be due to the rhizosphere modification, root releasing chemicals and alteration in the soil physio-chemical and enzymatic activities due to mix and different rooting behavior during intercropping (Nasar et al., 2022a). Nitrogen fertilization could also play an important role in improving plant N status, which might improve the soil N availability for plant roots (Yong et al., 2018; Ochieng’ et al., 2021). For example, barely in intercropping with fababean was reported to have considerably improved the N content and total N uptake of barely because of the N fixation ability of the companion fababean (Galanopoulou et al., 2019), which confirmed our results. Maize-common bean intercropping has also been shown to have enhanced N contents and its uptake in the maize crop particularly under optimal N fertilizer application (Malunga et al., 2018). Several other cereal-legume intercropping studies have shown to improve the N content and its uptake in cereal crops *via* underlying facilitative N transfer from legume side to their companion cereal crop, rhizosphere modification, soil nutrient availability improvement, root releasing chemicals, changes in the nutrients related soil enzymes and some unknown mechanisms (Zhang et al., 2014; Chen et al., 2017; Hu et al., 2018; Gao and Meng, 2020; Nyawade et al., 2020a; Shao et al., 2020; Nasar et al., 2022a).

The present study also demonstrated that maize-soybean intercropping significantly increased the N yield indices (i.e., grain N yield and residue N yield) and N use efficiency indices (i.e., NUE, PNUE, NUpE and NAE) of maize crop. However, these indices were further increased under N_1_ treatments than in N_0_ and N_2_ treatments. Probably, this might be due to the underlying facilitation, or complementary (Gitari et al., 2018b; Li et al., 2020), sharing of nutrients (Shao et al., 2020), better use of soil available N and the facilitative N transfer from legume to their companion cereals during intercropping (Yong et al., 2018; Nyawade et al., 2020a). Moreover, N fertilization help reduce the belowground interspecific competition and maximize the facilitative interactions for resources between intercrops (Xiao et al., 2013). As earlier reported that legumes in intercropping with cereals modify the rooting system of cereals, enabling them to occupy more space and acquire more nitrogen (Galanopoulou et al., 2019). Moreover, the facilitative N transfer from legumes to cereals can make a reverent contribution to the N nutrition of cereals (Génard et al., 2016; Zhang et al., 2017a). In previous maize-soybean intercropping study it was found that intercropping significantly improves N content, N uptake and N use efficiency of maize crop particularly under optimized N fertilization, mainly because of the underlying rhizosphere modification and nutrient facilitation provided by soybean (Yong et al., 2018). In another maize-soybean intercropping study it was reported that the significant N transfer from soybean to maize improved the NUE of maize when treated with optimal N fertilization (Zhang et al., 2017b; Raza et al., 2022).

Such improved N status and N use efficiency in the cereal-legume intercropping are directly linked to nitrogen assimilatory enzymes such as NR, NiR and GOGAT activities, which are the key enzymes involve in plant nitrogen metabolism (Nasar et al., 2022b). This study showed that maize-soybean intercropping significantly improved the NR, NiR and GOGAT activity of maize crop as compared with mono-cropping. However, these enzymes were more evident when intercropping was practiced under N_1_ treatments than N_0_ and N_2_ treatment. Possibly, this might be attributed to the nitrogen fixation ability of soybean, which helps improve the nitrogen content of maize plant, thereby enhancing the N metabolism and N-related enzymes of maize crop (Nasar et al., 2022a). It might also be due to the underlying rhizosphere alteration, changes in the soil enzymes and the root releasing chemicals, which ultimately triggers the plant nitrogen metabolisms system (Nasar et al., 2022b). Moreover, nitrogen fertilization is also known to improve the nitrogen metabolism of the plant by improving the nitrogen assimilatory enzymes (Ben, 2016). These results are also supported by Nasar et al. (2022a), who found that maize-soybean intercropping under optimal N fertilization significantly improved the N assimilatory enzymes of maize crop, thereby enhancing its nitrogen use efficiency. Dang et al. (2020) also reported that proso millet and mung bean intercropping significantly improved the nitrogen assimilatory enzymes of millet crops, thereby enhancing their N status and yield. This, suggests that maize-soybean intercropping under optimal N fertilization can help improve the N uptake, N yield and N use efficiency *via* regulating N assimilatory enzymes, thereby enhancing its productivity.

## 5 Conclusion

The findings of this study have clearly shown that maize-soybean intercropping significantly improved the yield and yield attribute of maize compared with a mono-cropping system. However, these indices were more pronounced under optimal nitrogen fertilization. Moreover, intercropping under optimal nitrogen fertilization enhanced the nitrogen assimilatory enzymes such as nitrate reductase, nitrite reductase and glutamate synthase activity. This resulted in an improved nitrogen content and total nitrogen uptake of maize crop, thereby enhancing its nitrogen yield indices and nitrogen utilization efficiency indices such as nitrogen use efficiency, partial factor nitrogen use efficiency, nitrogen uptake efficiency and nitrogen agronomic efficiency as compared with mono-cropping. Hence, our study suggests that maize-soybean intercropping could be a potential cropping system for improving crop productivity, nitrogen uptake, nitrogen yield and nitrogen use efficiency under minimal input, ultimately leading to sustainable agricultural development.

## Data availability statement

The raw data supporting the conclusions of this article will be made available by the authors, without undue reservation.

## Author contributions

JN: conceptualization, methodology, and writing—original draft. CZ and RK: data curation. HG and ZS: formal analysis. GA and IH: resources. ZI and WA: software. XZ and JY: supervision. HG, QL, and RR: writing— review and editing. All authors contributed to the article and approved the submitted version.
